# Overexpression of Poplar Pyrabactin Resistance-Like Abscisic Acid Receptors Promotes Abscisic Acid Sensitivity and Drought Resistance in Transgenic *Arabidopsis*

**DOI:** 10.1371/journal.pone.0168040

**Published:** 2016-12-19

**Authors:** Jingling Yu, Lei Yang, Xiaobing Liu, Renjie Tang, Yuan Wang, Haiman Ge, Mengting Wu, Jiang Zhang, Fugeng Zhao, Sheng Luan, Wenzhi Lan

**Affiliations:** 1 State Key Laboratory for Pharmaceutical Biotechnology, Nanjing University-Nanjing Forestry University Joint Institute for Plant Molecular Biology, College of Life Sciences, Nanjing University, Nanjing, China; 2 Department of Plant and Microbial Biology, University of California, Berkeley, California, United States of America; 3 Hubei Collaborative Innovation Center for Green Transformation of Bio-Resources, College of Life Sciences, Hubei University, Wuhan, China; National Taiwan University, TAIWAN

## Abstract

Drought stress is an important environmental factor limiting productivity of plants, especially fast growing species with high water consumption like poplar. Abscisic acid (ABA) is a phytohormone that positively regulates seed dormancy and drought resistance. The PYR1 (Pyrabactin Resistance 1)/ PYRL (PYR-Like)/ RCAR (Regulatory Component of ABA Receptor) (PYR/PYL/RCAR) ABA receptor family has been identified and widely characterized in *Arabidopsis thaliana*. However, their functions in poplars remain unknown. Here, we report that 2 of 14 PYR/PYL/RCAR orthologues in poplar (*Populus trichocarpa*) (PtPYRLs) function as a positive regulator of the ABA signal transduction pathway. The *Arabidopsis* transient expression and yeast two-hybrid assays showed the interaction among PtPYRL1 and PtPYRL5, a clade A protein phosphatase 2C, and a SnRK2, suggesting that a core signalling complex for ABA signaling pathway exists in poplars. Phenotypic analysis of *PtPYRL1* and *PtPYRL5* transgenic *Arabidopsis* showed that these two genes positively regulated the ABA responses during the seed germination. More importantly, the overexpression of *PtPYRL1* and *PtPYRL5* substantially improved ABA sensitivity and drought stress tolerance in transgenic plants. In summary, we comprehensively uncovered the properties of PtPYRL1 and PtPYRL5, which might be good target genes to genetically engineer drought-Resistant plants.

## Introduction

Abscisic acid (ABA) is a phytohormone that regulates key responses relevant to stresses such as biotic and abiotic stress. Upon abiotic stress conditions, especially drought, ABA biosynthesis is rapidly induced to initiate a number of molecular and cellular responses, among which the well-known are the expression of stress-related genes and stomatal closure, leading to adaptation to the stress conditions [1,2]. In addition, ABA also plays critical roles in many developmental stages including seed development, dormancy, germination, vegetative growth [3]. Thus, ABA is considered to be one of the most important phytohormones that contribute to the global agriculture.

ABA receptors are the first factor for initiating ABA signaling. Two independent research groups revealed a new type of soluble proteins, PYR1 (Pyrabactin Resistance 1) / PYRL (PYR-Like)/ RCAR (Regulatory Component of ABA Receptor) (hereafter referred to as PYR/PYL/RCAR for simplicity), as ABA receptors in *Arabidopsis thaliana* [4,5]. The *Arabidopsis* genome contains 14 *PYR/PYL/RCAR* genes, which encode highly conserved small proteins with 159–211 amino acid residues, belonging to a highly conserved family containing a steroidogenic acute regulatory-related lipid transfer (START) domain [4,5,6]. Four members, *PYR1*, *PYL1*, *PYL2* and *PYL4* impaired caused the quadruple mutant *Arabidopsis* lines displayed the strong ABA-insensitive phenotype [5], and this phenotype became stronger once *PYRL5* and *PYRL8* were also impaired [7]. Conversely, overexpression of *PYRL5* produces enhanced ABA responses in *Arabidopsis thaliana* [4]. Although solid studies revealed PYR/PYL/RCAR proteins played a critical role in ABA responses in *Arabidopsis thaliana*, there is little knowledge regarding molecular identity of these ABA receptors in poplars.

A protein phosphatase–kinase complex [type 2C protein phosphatase (PP2C)- SNF1-related protein kinase 2 (SnRK2)] functions as downstream components of PYR/PYL/RCARs [1,2]. Among the many PP2C family members in plants, clade A, consisting of nine members in *Arabidopsis*, are targets for PYR/PYL/RCAR receptors to bind [1,2,4,5]. In the absence of ABA, clade A PP2Cs physically bind to SnRK2s to dephosphorylate SnRK2s, resulting in the inhibition of ABA signal transduction, while in the presence of ABA, SnRK2s will be released from such inhibitory regulation by PP2C to activate their aims [1,4,5,6]. We have shown previously that PP2CA, a clade A PP2C member, lies upstream of the SnRK2-type kinases that, in turn, interact with and activate the anion channel SLAC1 to control the ABA response in guard cells [8,9]. Presently, the PYR/PYL/RCAR–PP2C–SnRK2 complex is acceptable for a core regulatory network in ABA responses in plants.

Being great economic and ecological importance in temperate regions, poplars are widely planted throughout the world, including drought environments. They show high productivity, but exhibit a strong demand for water [10]. Therefore, poplar growth is severely disrupted upon drought stress. As core members in ABA signaling, two orthologs of *Arabidopsis* clade A PP2Cs cloned from poplars reduced ABA sensitivity [11,12], and a SnRK2-type kinase *PtSnRK2*.*7* enhanced salt stress resistance in the transgenic lines [13], indicating the PP2C-SnRK2 complex might play critical roles in stress tolerance in poplars. However, their upstream proteins PYRLs in poplars are still unclear. Interestingly, overexpression of OsPYRL5, and several tomato PYR/PYL/RCARs enhanced the transgenic lines resistant to drought [14,15]. In this work, we overexpressed two *PYRL*s cloned from *Populus trichocarpa*, *PtPYRL1* or *PtPYRL5*, in *Arabidopsis* by *Agrobacterium*-mediated transformation. Our data indicate that transgenic plants enhanced ABA sensitivity with reduction of seed germination and improvement of drought resistance, providing a potential application of *PYRL* genes on engineering plants.

## Materials and Methods

### Plant materials and growth conditions

The ecotype of *Arabidopsis thaliana* and poplar used here is Columbia-0 (Col-0) and *Populus davidiana* × *Populus bolleana*, respectively. *Populus davidiana* × *Populus bolleana* is an ideal *Populus* species for the practical transgenic experiment due to its more stable traits compared with *Populus trichocarpa*. The poplars were amplified and kept by aseptically transferring shoot apices to solid Murashige and Skoog (MS) medium with 0.1 mg·L^−1^ naphthaleneacetic acid. The surface-sterilized *Arabidopsis thaliana* seeds were plated on MS medium containing 1% (w/v) sucrose and pH was adjusted to 5.7, and solidified using 0.8% (w/v) agar. Seedlings were grown in the chambers at 22–24°C with light intensity of 150 μmol·m^−2^·s^−1^ and 16:8 h light/dark photoperiods. For soil culture, 7-day-old *Arabidopsis* seedlings or 30-day-old poplar plantlets germinated on MS medium were carefully removed to the nutrient-rich soil. The soil cultured plants were grown in the greenhouse under 150 μmol·m^−2^·s^−1^ light intensity with a 16-h light/8-h dark photoperiod at 22°C.

### Phylogenetic analysis on PtPYRLs

To identify the candidate gene sequences encoding PYRLs from poplar plants, a blast search was conducted in the update poplar genome database (http://www.phytozome.net/poplar) based on the *Arabidopsis thaliana* PYR1 protein sequence (At4G17870.1) obtained from TAIR (http://www.arabidopsis.org/). Two fragments containing *PtPYRL1* or *PtPYRL5* open reading frame (ORF) were amplified by polymerase chain reaction (PCR) using leaf cDNA sample as template, and cloned into the SmaI/SalI or BamHI/SalI sites in a modified pCAMBIA1301 vector driven by 2×35S promoter, respectively. After sequence confirmation and manual revision of the coding sequence (CDS), amino acid sequence alignment of *Arabidopsis thaliana* and *P*. *trichocarpa* PYRLs (S1 Fig) was performed using the CLUSTALX program (http://www.clustal.org/), and a linearized neighbor-joining tree was produced with MEGA software version 4.0 [16].

### *PtPYRLs* expression measurement

For gene expression analysis, total RNA was extracted with the TRIzol reagent (Invitrogen) following the manufacturer’s instructions from various tissues (apex of shoot, mature leaf, stem, and root) of 3-month-old poplars (*Populus davidiana* × *Populus bolleana*) grown in the greenhouse. Two micrograms of total RNA was subjected to reverse transcription reaction using M-MLV reverse transcriptase (Promega) at 37°C for 1 h. The resultant cDNA was used for PCR amplification with the gene-specific primers (S1 Table).

Quantitative real-time PCR (qRT-PCR) was performed using the QuantiFast SYBR Green PCR Kit (Qiagen) on a CFX Connect Real-Time System (Bio-Rad). Each PCR mixture contained 10 μL 2× SYBR Green Master Mix, and 7 μL of RNA-free water (total of 20 μL). qRT-PCR was performed as follows: 94°C for 4 min followed by 40 cycles of 94°C for 15 s, 60°C for 30 s, and 72°C for 30 s. Primer specificity was confirmed by analysis of the melting curves. The 2^−ΔΔCT^ method [17] was used to quantify the value of every sample using *Actin2* or poplar *EF1β* as an internal reference. The PCR primers are listed in S1 Table.

### Subcellular localization studies

To determine the subcellular localization of PtPYRLs, PtABI1B and PtSnRK2.11, the coding region without a stop codon of *PtPYRL1*, *PtPYRL5*, *PtABI1B* or *PtSnRK2*.*11* was in-frame fused to the green fluorescent protein (GFP) sequence in the pEZS-NL plasmid and transferred into *Arabidopsis* mesophyll protoplasts by polyethylene glycol-mediated transfection [18]. After incubated for 16 h at 23°C, the protoplasts were imaged on GFP fluorescence by LSM 710 confocal microscope (Zeiss) equipped with an argon/krypton laser. The excitation wave length for GFP was 488 nm, and the emission signal was collected between 498 and 539 nm.

### Yeast two-hybrid assay

The full-length cDNA of *PtPYRL1*, *PtPYRL5*, and *PtSnRK2*.*11* were cloned into the DNA-binding domain (BD) vector pGBKT7, and PtABI1B was cloned into the activation domain (AD) vector pGADT7. The recombinant vectors were transformed into yeast strain AH109 by the lithium acetate method reported as previous [8]. Transformants were selected the synthetic complete agar medium (SC) minus leucine and tryptophan (SC/-Leu-Trp) and grown at 30°C for 4 d, and then transferred on SC minus leucine, tryptophan, and histidine, (SC/-Leu-Trp-His), or SC minus leucine, tryptophan, histidine, and adenine (SC/-Leu-Trp-His-Ade) supplemented with 20 mM 3-amino-1,2,4,-triazole (3-AT). For serial dilution assay, exponentially grown yeast cells were harvested and adjusted to OD_600_ = 0.5 with sterilized double-distilled water and diluted to 1, 1/10, 1/100, and 1/1000. Each yeast two-hybrid assay has been independently repeated at least three times. All primers used in this study are summarized in S1 Table.

### Generation of transgenic *Arabidopsis* lines

To generate plant transformation constructs, the full-length of *PtPYRL1* and *PtPYRL5* was cloned into a modified pCAMBIA-1301 vector via the *SmaI/SalI* or *BamHI/SalI* sites, respectively (S1 Table). The resultant plasmids were introduced into the *Agrobacterium tumefaciens* GV3101 strain for transformation into *Arabidopsis thaliana* (Col-0) by the floral dipping method [19]. Putative transgenic plants were screened on MS medium supplemented with 30 μg.L^−1^ hygromycin and then transferred to soil for propagation. Hygromycin-resistant plants of T2 generation were subjected to transgene expression analysis.

### Seed germination assay

After surface sterilization of the wild type and T3 transgenic *Arabidopsis* seeds, ~100 seeds were sown on MS plates without (control plates) or supplemented with 1 and 2 μM ABA. Seeds were incubated at 4°C for 3 days before being placed at the growth chambers with 22°C. The percentage of seeds that had germinated and developed fully green expanded cotyledons was determined, and was scored at the indicated time points.

### Drought treatments and transpiration rate assays

7-day-old seedlings grown at MS medium were transplanted in 50 g nutrient-rich soil saturated with water in 7.5-cm-width square pots and each pot contained 13 seedlings of uniform size. Drought treatment was performed in the greenhouse with regularly watering. Water was withheld from 10-day-old plants for 20 days before resuming watering. Photographs were taken for determining survival rate on the 3rd day after resuming watering.

To determine the transpiration rate, the whole rosettes were detached from 4-week-old seedlings grown in the greenhouse and then were placed in a weighing dish. Dishes were kept on the laboratory bench (21–22°C, approximately 40% relative humidity) for various time periods, and the loss of fresh weight was determined. All assays were performed in three independent experiments, and mean values and standard errors are presented.

### Determination of proline and malondialdehyde (MDA) contents

The rosette leaves detached from the seedlings 20 days after drought stress were sampled separately for the determination of proline or MDA content. The proline was extracted using the colorimetric assay [20] and its content was measured by UV-Visible Spectrophotometer (Biomate 3S, Thermo) at the absorbance at 520 nm. The level of MDA was determined in terms of thiobarbituric acid-reactive substances concentration reported previously [21]. Each experiment was repeated at least three times.

### Stomatal aperture assay

The leaves of same position from the rosette were detached from 3-week-old seedlings of wild type and transgenic lines. After incubated at the stomata open buffer (MES 10 mM, KCl 50 mM, CaCl_2_ 0.1 mM, pH 6.15) with abaxial leaf surface downward for 2 hours under light in the growth chamber, the leaves were moved to the stomata open buffer containing 0 or 20 μM ABA. After 2 h further incubation, adhesive plastic tapes were used to stick leaf lower epidermis and then to tear off epidermal strips. The epidermal strips stuck with adhesive tape were observed under the microscope (BX51, Olympus). At least 200 stomata apertures at random fields of epidermal strips were measured in each experiment. All experiments were repeated at least three times.

## Results

### PYR/PYL/RCAR orthologs in poplars

Our previous studies demonstrated that the PYR/PYL/RCAR members mediated the interactions of PP2C–SnRK2 phosphatase–kinase pairs between the slow type anion channel SLAC1 to regulate stomatal movements in *Arabidopsis* [8,9]. *Polulus trichocarpa* has 14 PYR/PYL/RCAR orthologs through a blast search against AtPYR1 protein (At4G17870.1) sequence in the *Populus* genome (http://www.phytozome.net/poplar) (S1 Fig). *PtPYRL* genes can be classified into three subclasses, based on the classification of *Arabidopsis PYR/PYL/RCARs* (S1 Fig). To probe the potential candidates of PtPYRLs playing vital functions on ABA signaling in *Populus*, we focused on PtPYRL1, as it shares the closest amino acid sequence to AtPYR1, an ABA receptor controlling growth, development and stress responses in *Arabidopsis thaliana* [5–7] (S1 Fig). We also selected PtPYRL5, the one has the lowest phylogenetic relationship between PtPYRL1, to clarify the molecular mechanisms and physiological functions of PtPYRL families (S1 Fig). The amino acid sequence of PtPYRL1 and PtPYRL5 respectively shares 66.51% and 43.18% sequence identity of AtPYR1, and PtPYRL1 has 48.39% identity with PtPYRL5.

### Expression pattern and subcellular location of *PtPYRL1* and *PtPYRL5*

**Fig 1:**
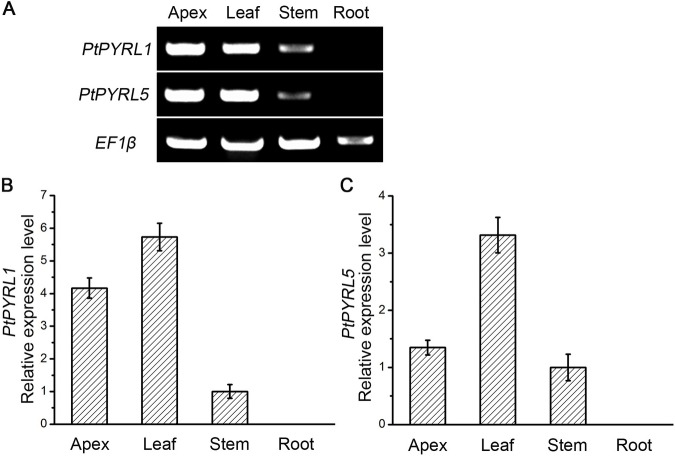
Expression pattern of *PtPYRL1* and *PtPYRL5* in tissues of poplar. (**A**) RT-PCR analysis of *PtPYRL1* and *PtPYRL5* mRNA levels in tissues of poplar. The apexes, leaves, shoots, and roots were from 3-month-old poplar grown in soil under long-day conditions. *EF1β* was used as a loading control. qRT-PCR analysis of *PtPYRL1* (**B**) and *PtPYRL5* (**C**) transcripts in various tissues of poplar. Total RNA was isolated from various tissues (apex, leaf, shoot, and root) of 3-month-old poplar. Values were normalized to *EF1β*, and the relative mRNA expression levels were calculated as the ratio of *PtPYRL1* or *PtPYRL1* mRNA level to *EF1β* level in corresponding tissue. Data are mean ± SD of four replicate experiments.

The members of PYR/PYL/RCAR family are preferentially expressed in some tissues to conduct distinct functions in plants [5–7]. To test expression patterns of *PtPYRL1* and *PtPYRL5***,** transcript abundance of these two genes was examined in various tissues in *Populus davidiana* × *Populus bolleana* by RT-PCR analysis. As shown in Fig 1A, both *PtPYRL1* and *PtPYRL5* were expressed strongly in the apexes and leaves, and had relative lower expression level in stems. Interestingly, *PtPYRL1* and *PtPYRL5* were rarely detected in the roots (Fig 1A). These results indicate that *PtPYRL1* and *PtPYRL5* displayed the similar expression patterns in *Populus*. We applied the quantitative Real-Time PCR (qRT-PCR) analysis to further confirm the expression patterns of these two *PtPYRLs*. Indeed, *PtPYRL1* and *PtPYRL5* were predominantly expressed in poplar apex and leaf tissues (Fig 1B and 1C). Specific tissues expression indicates that *PtPYRL1* and *PtPYRL5* might exert biological function major in the leaves.

**Fig 2:**
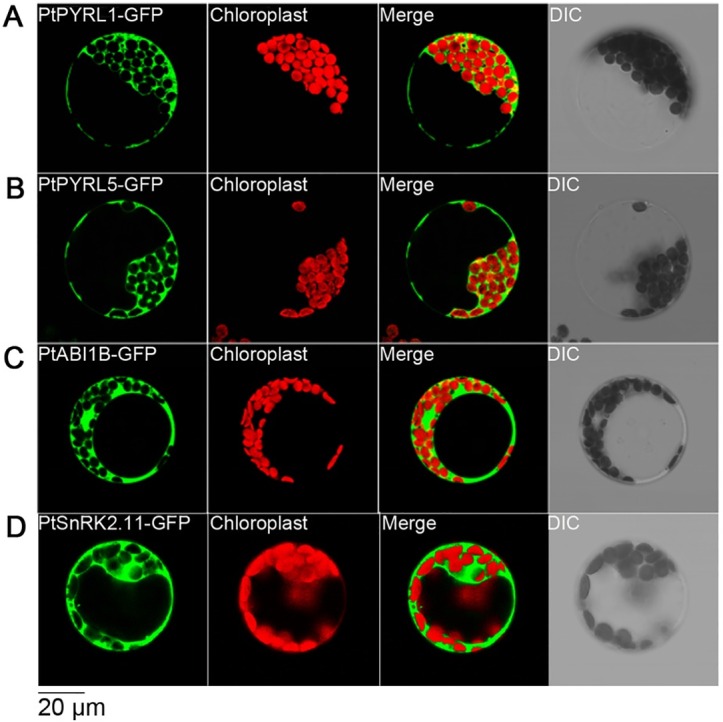
Subcellular localization of PtPYRL1-GFP, PtPYRL5-GFP, PtABI1B-GFP, and PtSnRK2.11-GFP in *Arabidopsis* cells. The GFP coding sequence was fused to the PtPYRL1, PtPYRL5, PtABI1B, and PtSnRK2.11 coding sequence without stop codon in the pEZS-NL vector driven by the 35S promoter, and then transformed into protoplasts of *Arabidopsis* mesophyll cells, respectively. The GFP signals of PtPYRL1-GFP (**A**), PtPYRL5-GFP (**B**), PtABI1B-GFP (**C**), and PtSnRK2.11-GFP (**D**) and chloroplast auto-fluorescence were observed by confocal microscopy, and displayed green and red colours, respectively. Overlay of both images was shown in the 3rd column. The panels in the 4th column were the respective bright-field images. Scale bar is 20 μm.

AtPYR1 is considered as a nucleocytoplasmic protein because that 35S::GFP-PYR1 protein in complemented transgenic *Arabidopsis* line is located in the cytoplasm and nucleoplasm [5]. To investigate whether PtPYRL1, the closest phylogenetic member to AtPYR1 in poplars, has the similar subcellular localization, the construct of 35S::PtPYRL1-GFP was transiently expressed into *Arabidopsis* mesophyll protoplasts. The fluorescence signals of GFP proteins were detected at the cytoplasmic part by confocal microscopic analysis (Fig 2A). We also tested the subcellular location of PtPYRL5 using the same method, and found that 35S::PtPYRL5-GFP also displayed the fluorescence signals at the cytoplasm (Fig 2B). The cytoplasmic location of PtPYRL1 and PtPYRL5 are consistent with the idea that ABA takes place at the cytoplasm to bind PYR/PYL/RCAR family members [1].

### Interaction of PtPYRLs-PtABI1B-PtSnRK2.11

**Fig 3:**
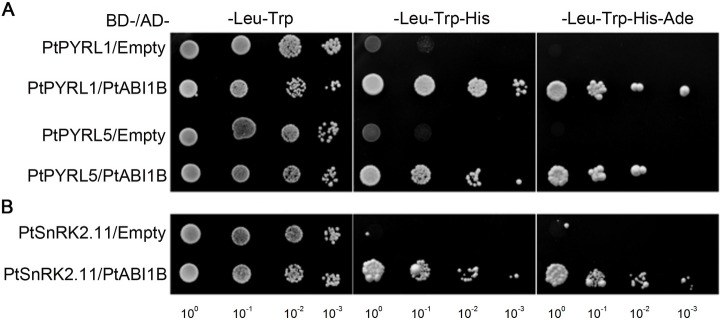
Yeast two-hybrid assay of the interactions of PtPYRL1-, or PtPYRL5-PtABI1B-PtSnRK2.11. The yeast strains AH109 were co-transformed with various BD and AD fusion constructs as indicated on the left of each row. Serial decimal dilutions of corresponding yeast cells were spotted on the synthetic complete agar medium (SC) minus leucine and tryptophan (-Leu-Trp; left lane), SC minus leucine, tryptophan and histidine (-Leu-Trp-His; middle lane), or SC minus leucine, tryptophan, histidine, and adenine (-Leu-Trp-His-Ade) containing 20 mM 3-AT (right lane). Photographs were taken after cultivation at 30°C for 4 days.

PYR/PYL/RCAR family members directly interact with clade A PP2Cs to down-stream ABA signaling [4,5]. ABI1 (ABA-insensitive 1) is one of PP2Cs member and acts as a key negative regulator of ABA stress signaling pathways in *Arabidopsis* [4,5,22], and its ortholog PtABI1B maintains the bud phenology and haplotype structure in *Populus* [23,24]. To examine whether PtPYRL1 and PtPYRL5 mediates ABA signaling through PtABI1B, we conducted an interaction assay by a yeast two-hybrid system, which successfully identified a number of interaction partners belonging to ABA signaling components in our previous studies [8,9]. Using PtPYRL1 and PtPYRL5 fused to the bait vector and PtABI1B in the activation vector, we cotransformed yeast cells with individual pairs and found that only yeast cells coexpressed PtPYRL1 and PtABI1B, or PtPYRL5 and PtABI1B could grow at the medium minus His, Leu and Trp, even the medium minus Ade, His, Leu and Trp supplemented with 3-AT (Fig 3A), indicating that PtPYRL1 and PtPYRL5 might physically interact with PtABI1B.

According to analyses of *Arabidopsis* genetics and the protein-protein interaction, ABI1 interacts with SnRK2 type kinases, including SnRK2.6, to control its ABA induced activity [1]. SnRK2.6, also named as OST1 (OPEN STOMATA 1) or SnRK2E, is rapidly activated by ABA and positively regulates stomatal closure in response to ABA [1,3,8]. Considering SnRK2.6/OST1 is the global and positive regulator of ABA signaling in *Arabidopsis* [1], we analyzed if its homologous protein at poplar, PtSnRK2.11 interacts with PtABI1B. To test the possibility, we cotransformed yeast cells with PtSnRK2.11, or a pair of PtSnRK2.11 and PtABI1B. Indeed, the cells cotransformed with PtSnRK2.11 and PtABI1B still grew in the medium minus multiple nutrient components, and the cells cotransformed with PtSnRK2.11 and the control activation vector did not (Fig 3B). Furthermore, in transiently transgenic *Arabidopsis* mesophyll protoplasts, fluorescence signals of PtABI1B-GFP and PtSnRK2.11-GFP were primarily detected at the cytoplasm, at which PtPYRL1-GFP and PtPYRL5-GFP was located (Fig 2). Taken together, PtPYRL1 and PtPYRL5, PtABI1B, and PtSnRK2.11 might constitute an ABA signaling pathway in *Populus*.

### Overexpression of PtPYRL1 or PtPYRL5 decreased germination rates in transgenic *Arabidopsis*

**Fig 4:**
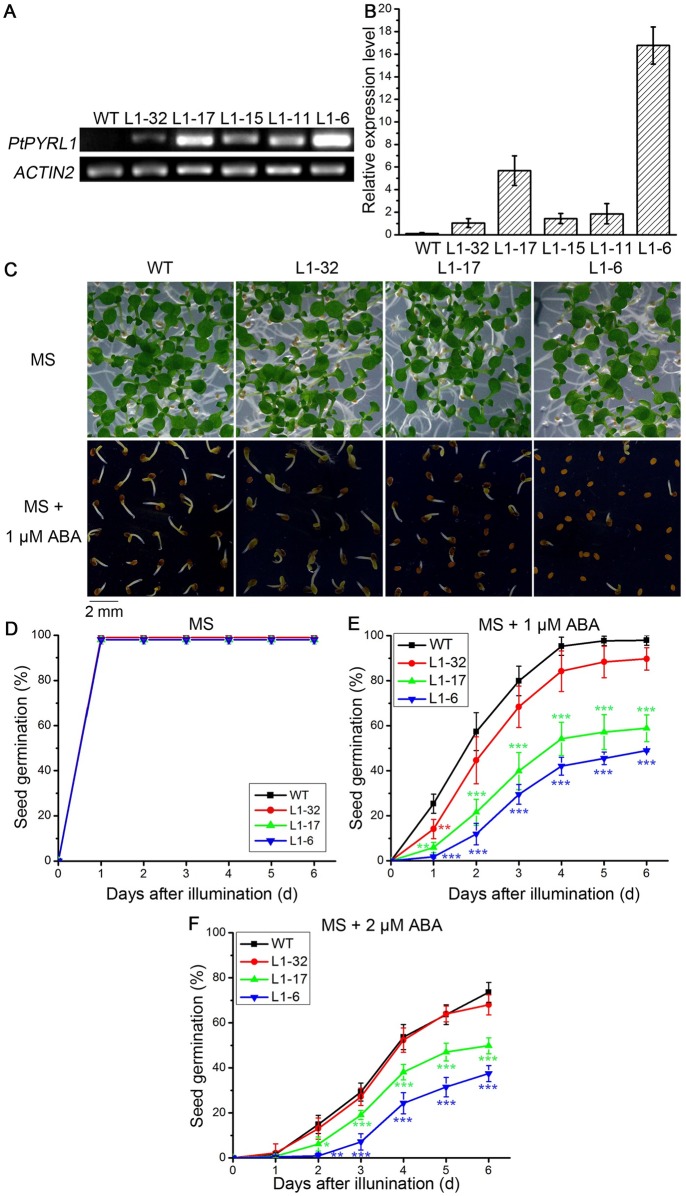
Overexpression of PtPYRL1 enhanced ABA sensitivity in the transgenic *Arabidopsis* plants. (**A**) Expression level of *PtPYRL1* in wild type (WT) and transgenic lines (L1-32, L1-17, L1-15, L1-11 and L1-6) grown at MS for 20 days. The level was detected using gene-specific primers after 26 PCR cycles. *ACTIN2* was used as endogenous control in all cDNA samples. (**B**) qRT-PCR analysis on *PYRL1* expression in wild type and transgenic seedlings. Total RNA was isolated from 20-day-old whole seedlings grown at MS. The relative expression level was calculated as the ratio of *PtPYRL1* level to *ACTIN2* level. (**C**) Representative images of 8-day-old wild type (WT) and transgenic lines (L1-32, L1-17, and L1-6) grown at the MS medium without (upper panel) or with 1μM ABA (low panel). The germination percentage of the seeds of wild type (WT) and transgenic lines (L1-32, L1-17, and L1-6) during growth at the MS medium containing 0 ABA (**D**), 1 μM ABA (**E**), or 2 μM ABA (**F**). Approximately 200 seeds were used in each experiment, and each experiment was repeated three times. Data are the standard deviation and significant differences are indicated as **P < 0.01 and ***P < 0.001 (ANOVA test).

To determine whether PtPYRL1 and PtPYRL5 contribute to the responses related to ABA signaling, we generated transgenic *Arabidopsis* overexpressing the coding sequence of *PtPYRL1* or *PtPYRL5* driven by a strong constitutive 35S promoter. To test whether these transgenic plants contained single- or multi-copy of transformed gene, we conducted Southern blot hybridizations using Hyg-specific probes. Transgenic *PtPYRL1* line L1-6 and *PtPYRL5* line L5-4 were contained single transgene integration, while transgenic line L1-32, L1-17, L1-11, L5-6, and L5-16 had multi-copies of the transgene (S2 Fig). RT-PCR assay was used to further analyze the effects of copy number of transformed genes on transcriptional levels in various transgenic *Arabidopsis* lines. Expression of *PtPYRL1* and *PtPYRL5* was undetectable in wild type plants (Fig 4A and S3A Fig). Among the transgenic lines, levels of *PtPRYL1* in line L1-6 and L1-17 were higher than those in lines L1-11, L1-15 and L1-32 (Fig 4A), and line L5-4 and L5-6 had high *PtPYRL5* expression while line L5-16 had weak PtPYRL5 expression (S3A Fig), displaying the transcriptional levels are inconsistent with the numbers of copy insertion of transgene. The expression levels of *PtPYRL1* and *PtPYRL5* in these transgenic lines were further confirmed by qRT-PCR assay. The relative *PtPYRL1* expression level of line L1-32, L1-17, L1-15, L1-11, and L1-6 was 1, 5.684, 1.442, 1.862, and 16.773, respectively (Fig 4B), and the relative *PtPYRL5* expression level of line L5-4, L5-6, and L5-16 was 5.2, 7.65, and 1, respectively (S3B Fig). These results suggested that the higher overexpression level in transgenic *PtPYRL1* or *PtPYRL5* lines might not result from the multi-copy insertion of transgene. Transgenic *Arabidopsis* lines with differential *PtPYRL1* expression levels, L1-6, L1-17, and L1-32, and the lines with differential *PtPYRL5* expression levels, L5-4, L5-6, and L5-16, were thus selected to perform physiological analysis in this study.

When grown on normal MS medium, transgenic lines L1-6, L1-17, L1-32, L5-4, L5-6, and L5-16 did not show significantly visible phenotypic changes. The germination and subsequent growth of these transgenic seedlings were similar to that of the wild type plants (Fig 4C and S3C Fig). To analyze the potential roles in seed germination, one of the most important physiological functions conducted by ABA in *planta*, wild type and three transgenic seeds were planted on MS medium supplemented with ABA. The germination greening ratio and germination rate of 8-day-old line L1-17, L1-6, L5-6, and L5-4 were significantly inhibited by 1 μM ABA, but the inhibition in line L1-32, L5-16 and the wild type was less severe (Fig 4C and S3C Fig). More detailed analyses of germination rates under different times of treatment were performed. Most wild type and transgenic lines seeds germinated one day after planted at MS medium without ABA (Fig 4D and S3D Fig), indicating these seeds were viable and energetic. After sowed in the medium containing 1 μM ABA, the germination of line L1-17 and L1-6 seeds was significantly delayed, and 60% and 50% of L1-17 and L1-6 seeds were germinated, respectively, while less than 95% of wild type or 90% of L1-32 seeds germinated (Fig 4E). Furthermore, the germination rates of line L1-17 and L1-6 seeds were further inhibited to 48% and 38%, respectively, and those of the wild type and L1-32 were not significantly different under 2 μM ABA conditions (Fig 4F). Similarly, the transgenic lines L5-6 and L5-4 with high *PtPYRL5* expression levels displayed more sensitive than the wild type and the line L5-16 with low *PtPYRL5* expression levels for ABA to inhibit seed germination (S3E and S3F Fig). These results showed that the transgenic lines with high expression level of *PtPYRL1* or *PtPYRL5* were more sensitive to ABA than those with low expression level of *PtPYRL1* or *PtPYRL5*, indicating that PtPYRL1 and PtPYRL5 appear to act as positive regulators in ABA-mediated seed germination inhibition.

### Overexpression of PtPYRL1 or PtPYRL5 enhanced drought resistance in transgenic *Arabidopsis*

**Fig 5:**
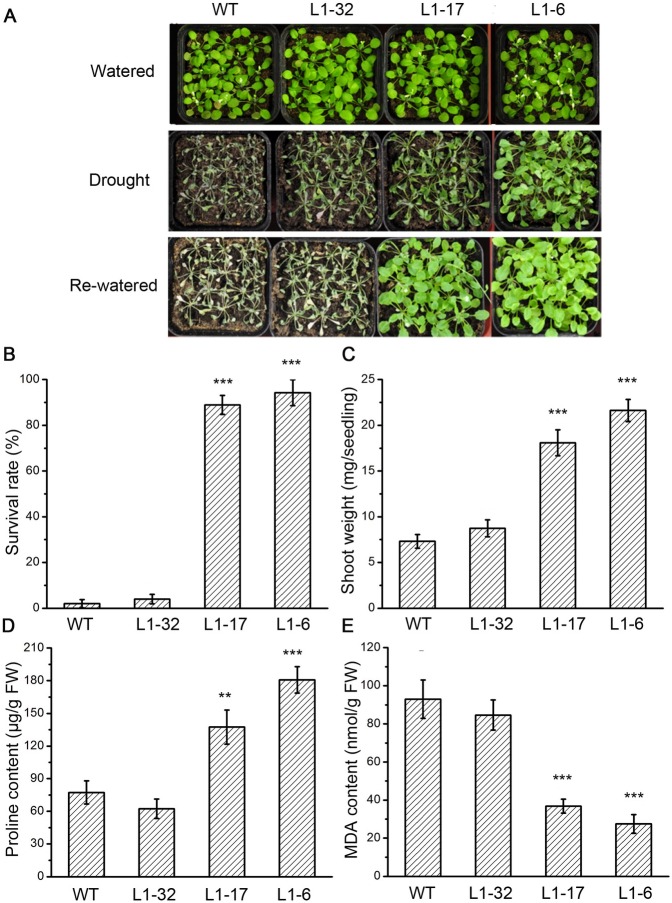
Overexpression of PtPRYL1 in *Arabidopsis* conferred drought resistance. (**A**) Representative images of wild type and transgenic PtPRYL1 lines under drought stress. The wild type (WT) and transgenic lines (L1-32, L1-17 and L1-6) were grown in soil with sufficient water for 10 days (Watered) (upper panel), and then water was withheld for 20 days before re-watered (Drought) (middle panel). Plants were then allowed to recover by watered again for 3 d before taking pictures (Re-watered) (low panel). (**B**) Quantification of the survival rate of the wild type (WT) and transgenic lines (L1-32, L1-17 and L1-6) at the third day after rewatering. Survival rates and standard deviations were calculated from the results of three independent experiments. (**C**) Quantification of shoot weight of the wild type (WT) and transgenic lines (L1-32, L1-17 and L1-6) at the third day after rewatering. Values are means ± SD (n = 26). Proline content (**D**) and MDA level (**E**) in the wild type (WT) and transgenic lines (L1-32, L1-17 and L1-6) after drought treatment for 20 days. Results are presented as means ± SD from three independent experiments.

Enhancement of drought resistance is another criterion in evaluating the ABA sensitivity of plants [1,2].To assess whether the drought resistance was altered in transgenic *PtPYRLs* lines, the wild type and lines of *Arabidopsis* overexpressing *PtPYRL1* (L1-32, L1-17 and L1-6) or *PtPYRL5* (L5-16, L5-6 and L5-4) were grown for 10 days in soil and then subjected to water withholding for an additional 20 days. At this time, the wild type, line L1-32 seedlings displayed obvious drought-stressed phenotypes, such as leaf wilting and senescence, whereas the line L1-6 plants were still turgid and their leaves remained green (Fig 5A). When the plants were rewatered and allowed to recover for 3 days, almost all the wild type and L1-32 plants were dead, but the line L1-6 and L1-17 plants survived (Fig 5A). Quantitative analysis found that 95% and 90% of the line L1-6 and L1-17 plants survived the treatment, respectively, while few of the wild type and L1-32 did (Fig 5B), and that the line L1-6 and L1-17 seedlings had more shoot weight than the wild type and L1-32 plants (Fig 5C). Similarly, the transgenic lines L5-6 and L5-4 with high *PtPYRL5* expression levels enhanced *Arabidopsis* survival and shoot weight under drought stress compared with the wild type and transgenic line L5-16 with low *PtPYRL5* expression level (S4A–S4C Fig).

To investigate the drought resistance mechanism, we examined proline accumulation and MDA formation, two processes involving in drought resistance capacity in plants [25,26]. Compared to the wild type, the line L1-6 and L1-17 plants with high *PtPYRL1* expression contained more proline and less MDA, whereas the line L1-32 plants with low *PtPYRL1* expression had similar proline and MDA (Fig 5D and 5E). Similarly, the lines L5-6 and L5-4 with high *PtPYRL5* expression level had more proline and less MDA than the wild type and the line L5-16 with low *PtPYRL5* expression level (S4D and S4E Fig). These results suggest that overexpression of *PtPYRL1* and *PtPYRL5* caused the increase in proline accumulation or the decrease in MDA formation to favor the drought resistance in plants.

### Overexpression of PtPYRL1 or PtPYRL5 decreased water loss in transgenic *Arabidopsis*

**Fig 6:**
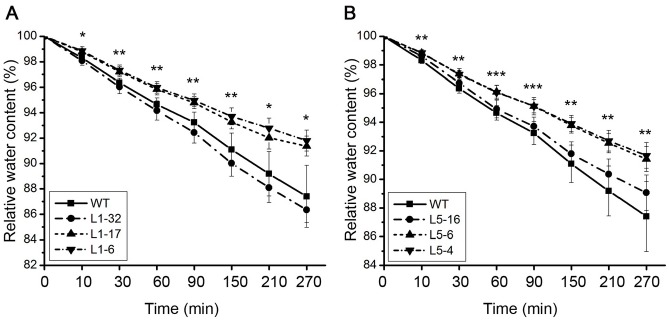
Water content of detached leaves from wild type and transgenic lines. (**A**) Comparison of the relative water content of leaves of wild type (WT) and transgenic *PtPYRL1* seedlings (L1-32, L1-17, and L1-6) at different time point after detached. Error bars indicate SD (n = 4). (**B**) Comparison of the rate of water loss of leaves of wild type (WT) and transgenic *PtPYRL5* seedlings (L5-16, L5-6, and L5-4) at different time point after detached. Error bars indicate SD (n = 4). Significant differences are indicated as *P < 0.05, **P < 0.01 and ***P < 0.001 (ANOVA test), respectively.

The transgenic plants with high expression levels of *PtPYRL1* or *PtPYRL5* became less wilted as compared to wild type plants under drought stress (Fig 5 and S4 Fig), suggesting a decreased transpiration rate in these transgenic plants. We thus determined the transpiration rate by measuring water loss from detached rosette leaves from the 30-day-old seedlings supplied with sufficient water. Once exposed to the air, the leaves detached from the wild type and transgenic line L1-32 plants displayed the gradual acceleration in water loss, while the rate of water loss in those from the transgenic lines L1-6 and L1-17 with high *PtPYRL1* expression levels was much slower (Fig 6A). Similarly, the rate of water loss in the leaves detached from the lines L5-6 and L5-4 with high *PtPYRL5* expression level was less than those in the wild type and the transgenic line L5-16 with low *PtPYRL5* expression level (Fig 6B). Therefore, the greatly decreased transpiration rate of leaves of the transgenic lines with high expression of *PtPYRL1* and *PtPYRL5* might enhance *Arabidopsis* drought avoidance.

### Overexpression of PtPYRL1 or PtPYRL5 promoted stomatal closure induced by ABA in transgenic *Arabidopsis*

**Fig 7:**
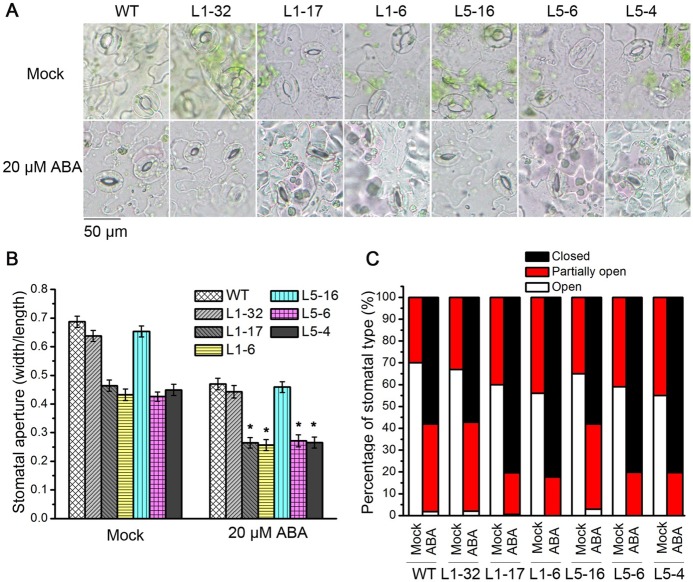
Stomatal movement in wild type, transgenic *PtPYRL1* or *PtPYRL5* seedlings under ABA treatment. **(A)** The representative images of stomata shape of wild type and transgenic seedlings in the presence or absence of ABA. The 3-week-old wild type (WT) and transgenic seedlings, L1-32, L1-17, L1-6, L5-16, L5-6, and L5-4 were treated with stomata open buffer (mock) (upper panel), or with 20 μM ABA (lower panel). Pictures were taken 2h after ABA treatment in Olympus BX51 microscope in 40x magnification. Scale bar is 50 μm. **(B)** Stomatal aperture of wild type and transgenic seedlings at 2h after treated with stomata open buffer (mock) or with 20 μM ABA. Stomatal aperture was calculated as the ratio of stomatal width to length. About 200 stomata were analyzed for each genotype. Data are the standard deviation and significant differences are indicated as *P < 0.05 (ANOVA test). (**C**) Percentage of stomatal status in wild type (WT) and transgenic seedlings at 2h after treated with stomata open buffer (mock) or with 20 μM ABA. Accordingly to the ratio of stomatal width to length, open status is 0.5–1.0, partially open status is 0.25–0.5, and closed status is < 0.25, respectively. Percentage of stomata was calculated based on the average of three independent replicate experiments.

To determine whether lower levels of transpiration rate resulted from ABA hypersensitivity in transgenic plants, we performed stomatal assays, and found that a significant difference of stomatal aperture between the wild type and the transgenic plants under the treatment with 20 μM ABA (Fig 7A). Stomatal apertures in the transgenic lines L1-6, L1-17, L5-4, and L5-6 reduced 41%, 43%, 41% and 36%, respectively, upon treatment with 20 μM ABA. However, the stomatal apertures in the wild type, L1-32, and L5-16 reduced 32%, 30%, and 31%, respectively (Fig 7B). We noticed that not all stomata were closed upon ABA treatment, and then divided stomatal status into three levels according to the ratio of stomatal width to length, open (0.5–1.0), partially open (0.25–0.5), and closed (< 0.25). To further test the sensitivity of stomatal movement in response to exogenous ABA, we compared the stomatal status in the wild type and transgenic *Arabidopsis*. As shown in Fig 7C, 80% and 20% of the stomatal of the transgenic lines L1-6, L1-17, L5-6, and L5-4 were at the closed and partially open status, respectively, while the wild type, L1-32, and L5-16 respectively had 60% closed stomata and 40% open stomata, providing further evidence that transgenic lines with high expression of *PtPYRL1* and *PtPYRL5* increase ABA sensitivity.

## Discussion

The functions of PYR/PYL/RCARs have been extensively explored in various plant species since they were identified as ABA receptors, especially in *Arabidopsis* [1]. Little information existed about the role of poplar PtPYRLs in mediating responses to diverse endogenous and environmental cues. In the present study, we provide evidence that putative PYR/PYL/RCARs exist in poplars and might function on the responses mediated by ABA. This finding revealed ABA sensitive and drought resistant properties of two PYRLs that might have biotechnological implications in engineering stress resistant plants.

*PYR/PYL/RCARs* encode proteins homologous to the START superfamily, sharing a conserved hydrophobic ligand–binding pocket [6,27]. As ABA receptors, PYR/PYL/RCARs at plants seem to be highly correlated with the evolution from green algae, mosses and ferns to angiosperms. Though aquatic algae do not have PYR/PYL/RCARs, PYR/PYL/RCAR family members of bryophyte, lycophyte and angiosperm, represented respectively with *Physcomitrella patens*, *Selaginella moellendorffii* and *Arabidopsis thaliana*, increase from 4, 10, to 14 [28]. We used BLAST research analysis based on the poplar database and found 14 PYRLs family members in poplars (S1 Fig), and exhibited these members could be grouped in three subfamilies that correlated well with corresponding *Arabidopsis* subfamilies. Diverse distribution of PYR/PYL/RCARs family members in different tissues is one of the critical ways for land plants to achieve adaption to the environments [1,15,28]. *PtPYRL1* and *PtPYRL5* showed the predominant transcription level in apex and leaf tissues, whereas little level in roots of poplars (Fig 1), therefore they are candidate genes to regulate ABA functions in aerial tissues.

*AtPYR/PYL/RCARs* encode a type of soluble proteins mostly localized to the cytosol, and deliver ABA in a regulated fashion to initiate rapid and controlled responses to various stress conditions [4,5]. Here, we also found that PtPYRL1 and PtPYRL5 are localized to the cytosol as fluorescence signals of PtPYRL1-GFP and PtPYRL5-GFP were specifically detected the cytoplasm of *Arabidopsis* mesophyll protoplasts (Fig 2A). Once cytosolic ABA is induced by environmental conditions or developmental cues, PYR/PYL/RCARs will bind ABA, and then interact with PP2C and inhibit its phosphatase activity [4,5]. Interestingly, PYR/PYL/RCARs may interact with specific PP2C members with or without ABA supplementation [4,5,9]. Our yeast two-hybrid assay showed that PtPYRL1 and PtPYRL5 could interact with PtABI1B in the absence of ABA (Fig 3), indicating that these two PtPYRLs exhibited high-affinity with PtABI1B. PtABI1B also interacted with PtSnRK2.11 (Fig 3), a protein kinase response for salt stress resistance [13]. Furthermore, PtSnRK2.11 and PtABI1B were localized to the cytosol, the same subcellular location as PtPYRL1 and PtPYRL5 (Fig 2). SnRK2s will be released from negative regulation by PP2Cs once PP2Cs interact with the ABA-bound PYR/PYL/RCARs, to phosphorylate the downstream facts [4,5,9]. Therefore, we speculate that PtPYRL1 and PtPYRL5, PtABI1B, and PtSnRK2.11 might form functional ABA signaling pathways in poplars.

Considering that numerous PYRLs in poplar, we preferred the overexpression method instead of constructing knock-down or knock-out lines to avoid the defect of mutation analysis that may be caused by functional redundancy. Overexpression of some PYR/PYL/RCARs from plants, including *Arabidopsis* [4,29], rice [14], and tomato [15] was shown to enhance ABA response and plant drought resistance. Here, the transgenic *Arabidopsis* lines highly overexpressed with *PtPYRL1* and *PtPYRL5* in *Arabidopsis* showed an ABA-hypersensitive phenotype, including enhancement of the negative effects of ABA on seed germination (Fig 4 and S3 Fig), as well as the increase in the positive effects that ABA signaling normally has under drought stress (Fig 5 and S4 Fig), including transpiration (Fig 6) and stomatal closure (Fig 7). All these results indicate that PtPYRL1 and PtPYRL5 function as positive regulators in ABA signaling. However, whether these functions of PtPYRL1 and PtPYRL5 result from interacting PtABI1B and PtSnRK2.11 remains further study.

In conclusion, data presented in this study demonstrate that poplars have multiple PYRLs, and constitutive overexpression of *PtPYRL1* and *PtPYRL5* in *Arabidopsis* increased ABA sensitivity. Overexpression of *PtPYRL1* and *PtPYRL5* enhanced proline accumulation and stomatal closure but reduced MDA formation and transpiration rate in transgenic plants, leading to improving drought resistance in these plants. Engineered ABA receptors have the possibility to improve plant yield or any other properties under advance abiotic stress. The substantially increased resistance to drought stress reported here might provide a potential strategy for engineering drought resistant plants by constitutively expressing the *PtPYRL1* and *PtPYRL5* genes.

## Supporting Information

S1 Fig Phylogenetic tree of PYRL receptors of *Populus trichocarpa* and *Arabidopsis thaliana*. The alignment of sequences among PYRLs was performed using the CLUSTALX program (http://www.clustal.org), and a linearized neighbor-joining tree was produced with MEGA software version 4.0. PYRL proteins of *Populus trichocarpa* are distributed in three major subfamilies.
(TIF)

S2 Fig Southern blot analysis of representative transgenic *Arabidopsis* plants. Line L1-32, L1-17, L1-11 and L1-6 represented the transgenic *Arabidopsis* plants overexpressing *PtPYRL1*; Line L5-4, L5-6 and L5-16 represented the transgenic *Arabidopsis* plants overexpressing *PtPYRL5*; WT, wide type of *Arabidopsis*. Genomic DNA (15 μg) from each transgenic plant was digested with BamHI, and then probed with the *Hyg* gene.
(TIF)

S3 Fig Overexpression of *PtPYRL5* enhanced ABA sensitivity in the transgenic *Arabidopsis* plants. (**A**) Expression level of *PtPYRL5* in wild type (WT) and transgenic lines (L5-16, L5-6, and L5-4) grown at MS for 20 days. The level was detected using gene-specific primers after 26 PCR cycles. *ACTIN2* was used as endogenous control in all cDNA samples. (**B**) qRT-PCR analysis on *PYRL5* expression in wild type (WT) and transgenic seedlings. Total RNA was isolated from 20-day-old whole seedlings grown at MS. The relative expression level was calculated as the ratio of *PtPYRL5* level to *ACTIN2* level. (**C**) Representative images of 8-day-old wild type (WT) and transgenic lines (L5-16, L5-6 and L5-4) grown at the MS medium without (upper panel) or with 1μM ABA (low panel). The germination percentage of the seeds of wild type (WT) and transgenic lines (L5-16, L5-6 and L5-4) during growth at the MS medium containing 0 μM (**D**), 1 μM (**E**), or 2 μM (**F**) ABA. Approximately 200 seeds were used in each experiment, and each experiment was repeated three times. Error bars represent the standard deviation with *P < 0.05 (Student’s t test), **P < 0.01 (Student’s t test), and ***P < 0.001 (Student’s t test).
(TIF)

S4 Fig Overexpression of *PtPRYL5* in *Arabidopsis* conferred drought resistance. (**A**) Representative images of wild type and transgenic PtPRYL5 lines under drought stress. The wild type (WT) and transgenic lines (L5-16, L5-6, and L5-4) were grown in soil with sufficient water for 10 days (Watered) (upper panel), and then water was withheld for 20 days before re-watering (Drought) (middle panel). Plants were then allowed to recover for 3 d before taking pictures (Re-watered) (low panel). (**B**) Quantification of the survival rate of the wild type (WT) and transgenic lines (L5-16, L5-6, and L5-4) at the third day after rewatering. Survival rates and standard deviations were calculated from the results of three independent experiments. (**C**) Quantification of shoot weight of the wild type (WT) and transgenic lines (L5-16, L5-6, and L5-4) at the third day after re-watered. Values are means ± SD (n = 26). Proline content (**D**) and MDA level (**E**) in the wild type (WT) and transgenic lines (L5-16, L5-6, and L5-4) after drought treatment for 20 days. Results are presented as means ± SD from three independent experiments. *P < 0.05 (Student’s t test); ***P <0.001 (Student’s t test).
(TIF)

S1 Table List of primers used in this study. (PDF)
