# Supplementary figures and images for: Overexpression of Poplar Pyrabactin Resistance-Like Abscisic Acid Receptors Promotes Abscisic Acid Sensitivity and Drought Resistance in Transgenic Arabidopsis
Source: PLoS One. 2016 Dec 19;11(12):e0168040. doi: 10.1371/journal.pone.0168040 (PMC5167274; doi:10.1371/journal.pone.0168040)

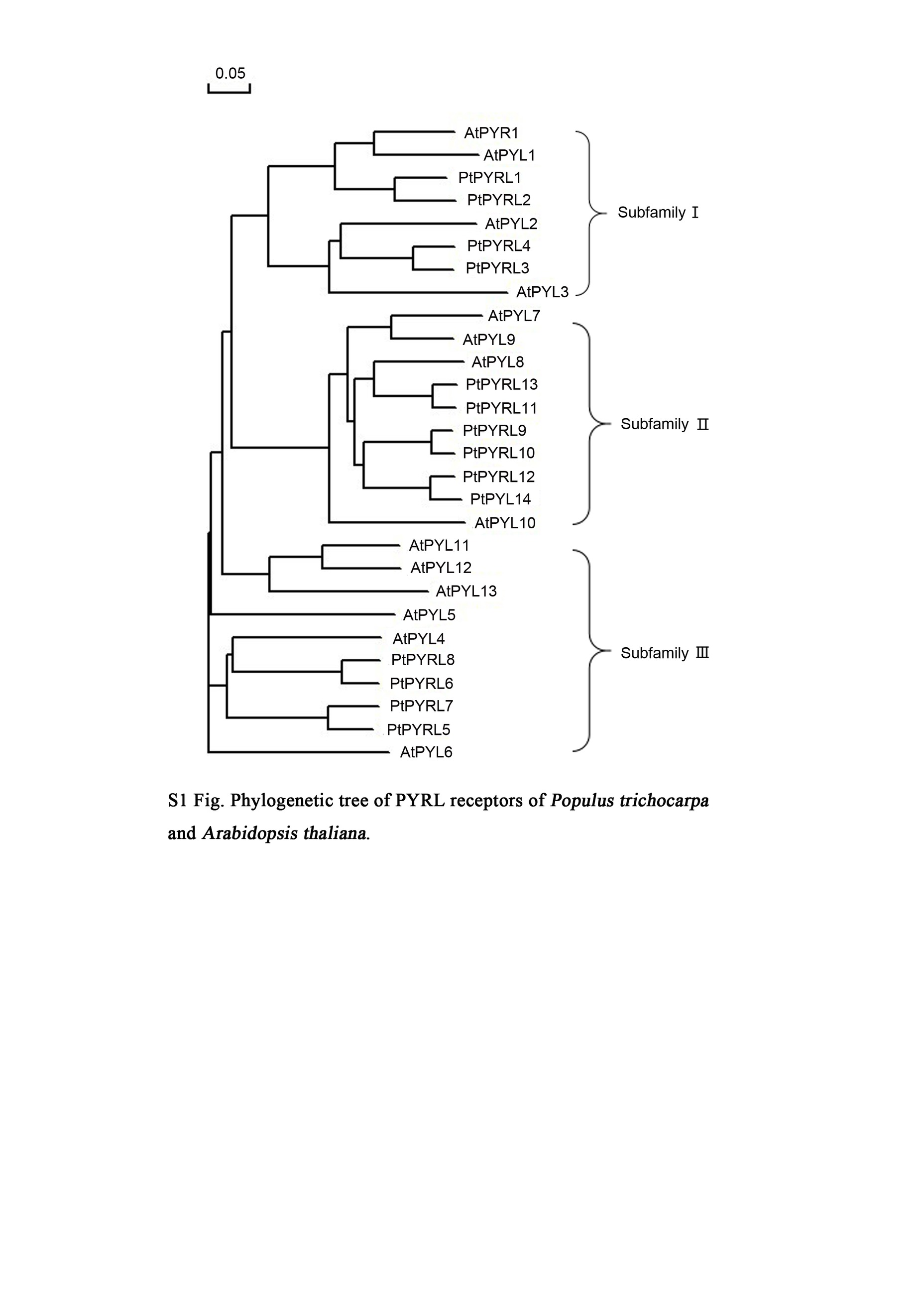

Supplement: S1 Fig — The alignment of sequences among PYRLs was performed using the CLUSTALX program (http://www.clustal.org), and a linearized neighbor-joining tree was produced with MEGA software version 4.0. PYRL proteins of Populus trichocarpa are distributed in three major subfamilies. (TIF) [file pone.0168040.s001.tif]

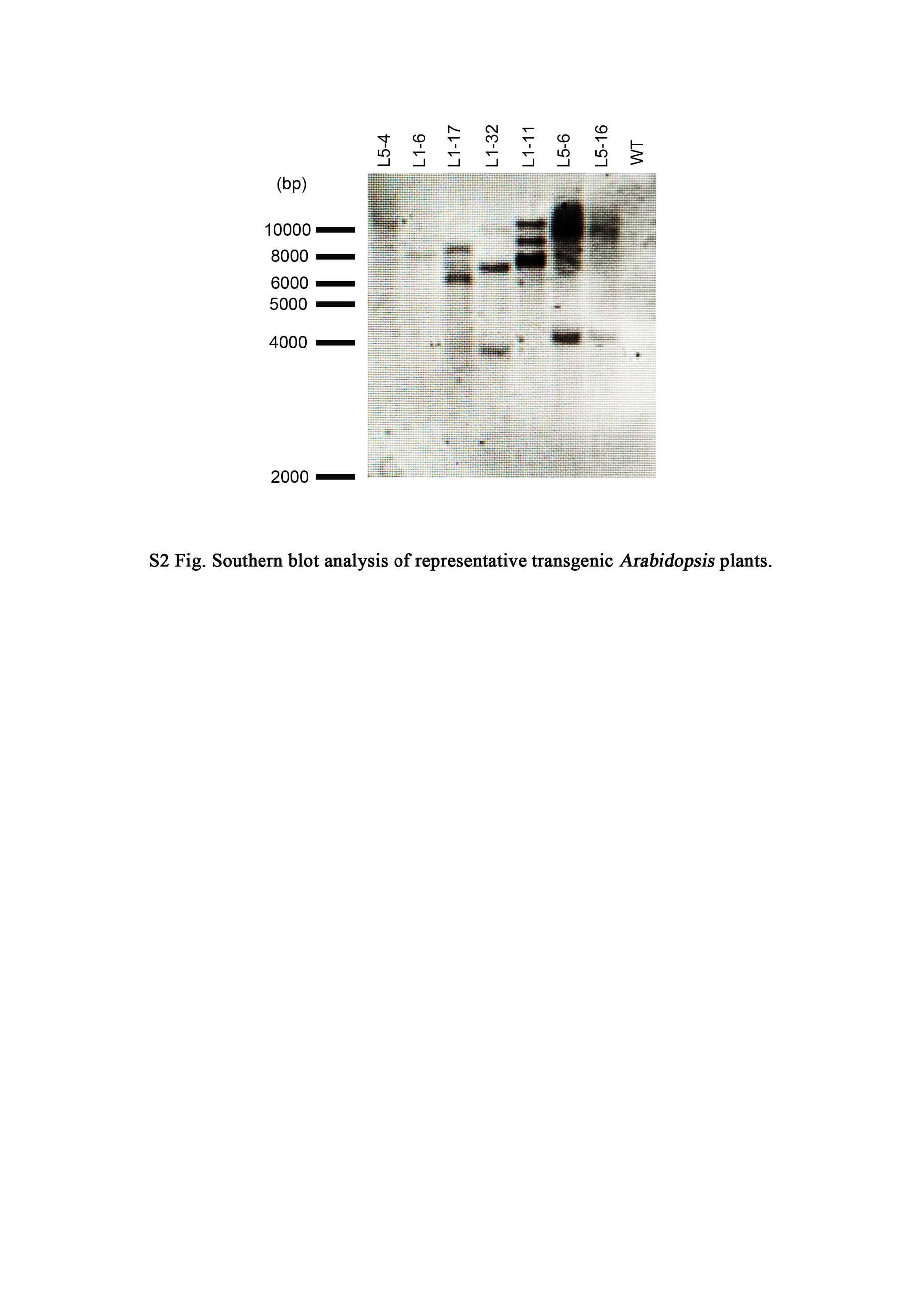

Supplement: S2 Fig — Line L1-32, L1-17, L1-11 and L1-6 represented the transgenic Arabidopsis plants overexpressing PtPYRL1; Line L5-4, L5-6 and L5-16 represented the transgenic Arabidopsis plants overexpressing PtPYRL5; WT, wide type of Arabidopsis. Genomic DNA (15 μg) from each transgenic plant was digested with BamHI, and then probed with the Hyg gene. (TIF) [file pone.0168040.s002.tif]

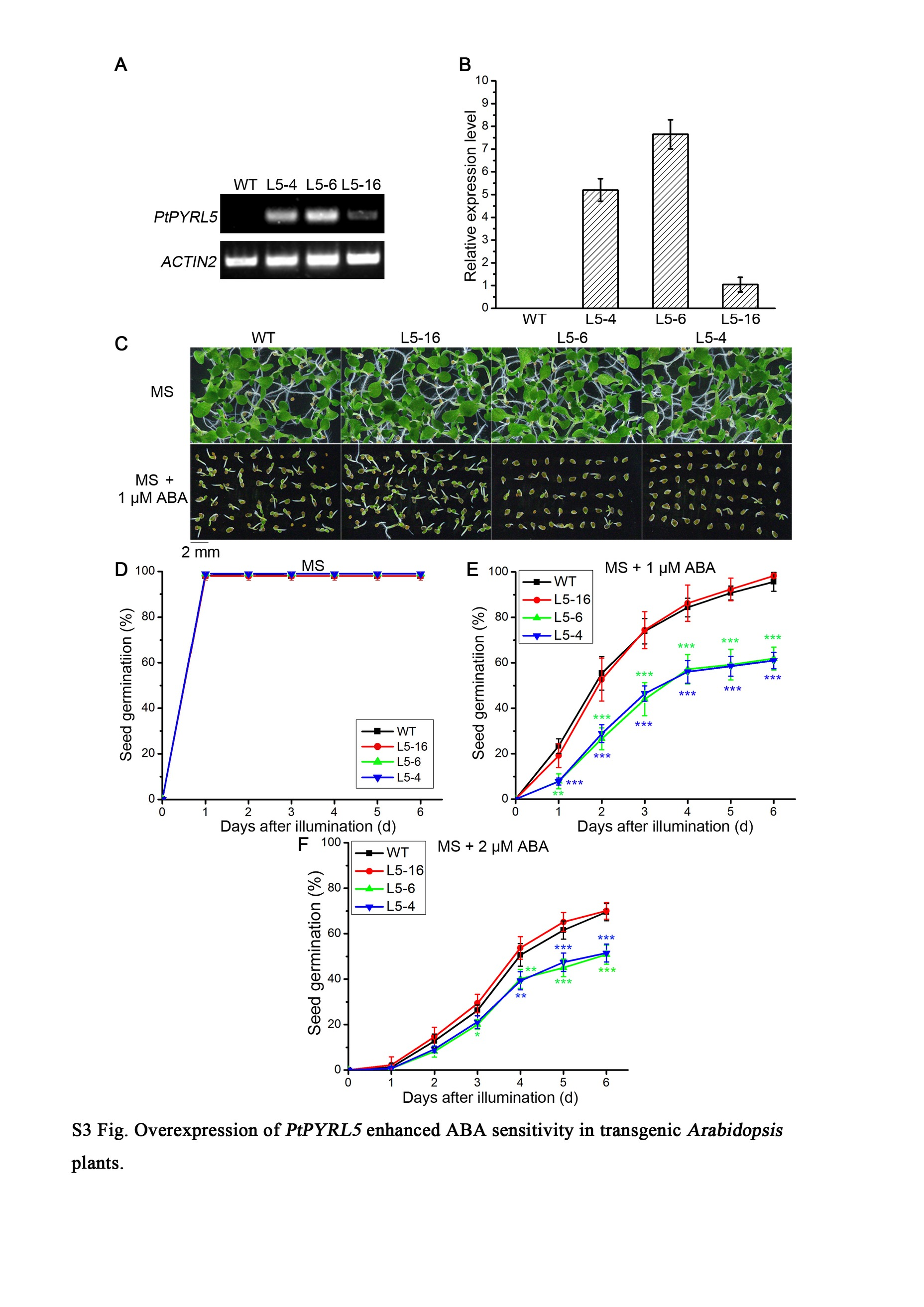

Supplement: S3 Fig — (A) Expression level of PtPYRL5 in wild type (WT) and transgenic lines (L5-16, L5-6, and L5-4) grown at MS for 20 days. The level was detected using gene-specific primers after 26 PCR cycles. ACTIN2 was used as endogenous control in all cDNA samples. (B) qRT-PCR analysis on PYRL5 expression in wild type (WT) and transgenic seedlings. Total RNA was isolated from 20-day-old whole seedlings grown at MS. The relative expression level was calculated as the ratio of PtPYRL5 level to ACTIN2 level. (C) Representative images of 8-day-old wild type (WT) and transgenic lines (L5-16, L5-6 and L5-4) grown at the MS medium without (upper panel) or with 1μM ABA (low panel). The germination percentage of the seeds of wild type (WT) and transgenic lines (L5-16, L5-6 and L5-4) during growth at the MS medium containing 0 μM (D), 1 μM (E), or 2 μM (F) ABA. Approximately 200 seeds were used in each experiment, and each experiment was repeated three times. Error bars represent the standard deviation with *P < 0.05 (Student’s t test), **P < 0.01 (Student’s t test), and ***P < 0.001 (Student’s t test). (TIF) [file pone.0168040.s003.tif]

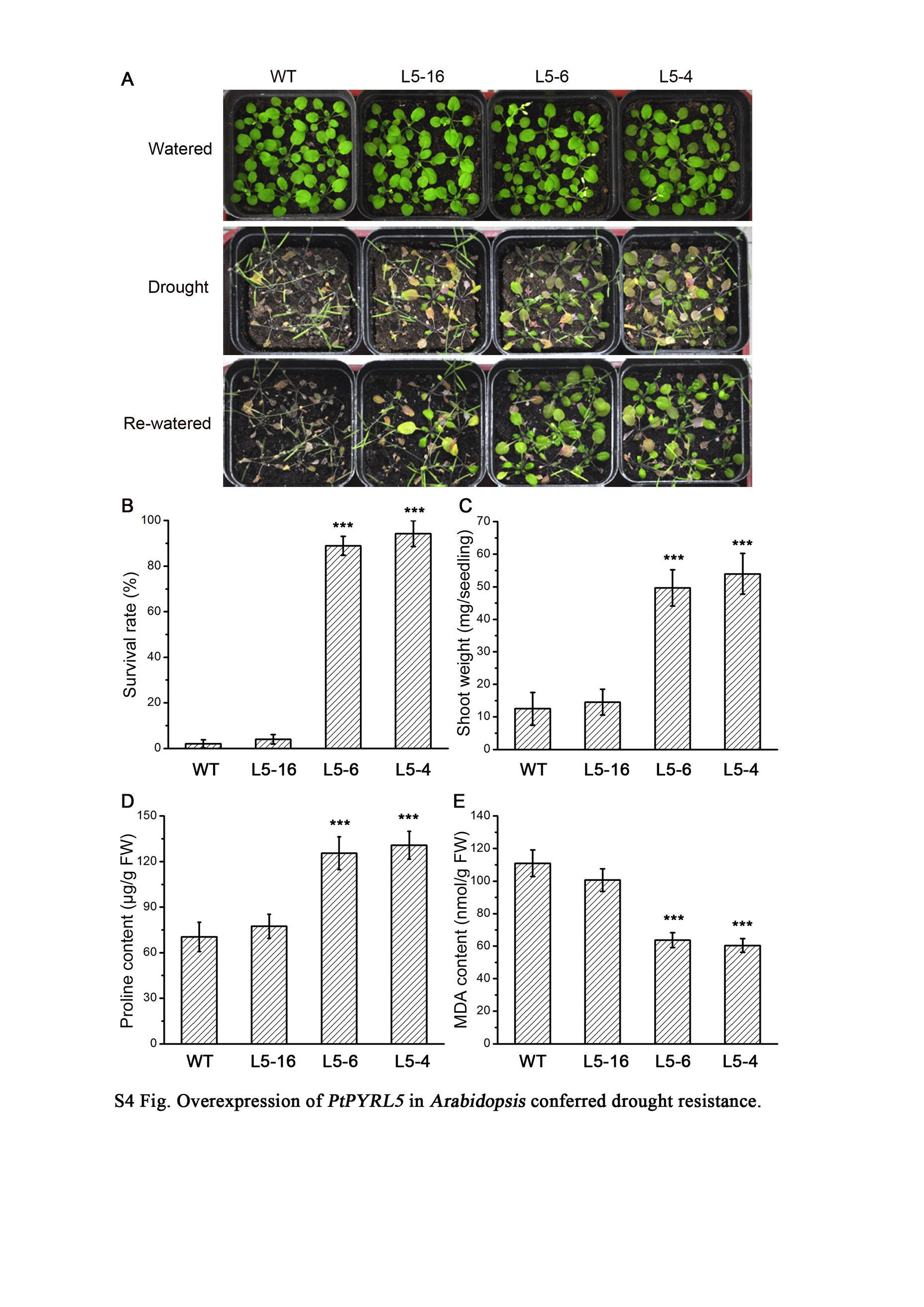

Supplement: S4 Fig — (A) Representative images of wild type and transgenic PtPRYL5 lines under drought stress. The wild type (WT) and transgenic lines (L5-16, L5-6, and L5-4) were grown in soil with sufficient water for 10 days (Watered) (upper panel), and then water was withheld for 20 days before re-watering (Drought) (middle panel). Plants were then allowed to recover for 3 d before taking pictures (Re-watered) (low panel). (B) Quantification of the survival rate of the wild type (WT) and transgenic lines (L5-16, L5-6, and L5-4) at the third day after rewatering. Survival rates and standard deviations were calculated from the results of three independent experiments. (C) Quantification of shoot weight of the wild type (WT) and transgenic lines (L5-16, L5-6, and L5-4) at the third day after re-watered. Values are means ± SD (n = 26). Proline content (D) and MDA level (E) in the wild type (WT) and transgenic lines (L5-16, L5-6, and L5-4) after drought treatment for 20 days. Results are presented as means ± SD from three independent experiments. *P < 0.05 (Student’s t test); ***P <0.001 (Student’s t test). (TIF) [file pone.0168040.s004.tif]
