# Supplementary material for: Overexpression of Poplar Pyrabactin Resistance-Like Abscisic Acid Receptors Promotes Abscisic Acid Sensitivity and Drought Resistance in Transgenic Arabidopsis
Source: PLoS One. 2016 Dec 19;11(12):e0168040. doi: 10.1371/journal.pone.0168040 (PMC5167274; doi:10.1371/journal.pone.0168040)
Supplement: S1 Table — (PDF) [file pone.0168040.s005.pdf]

S1 Table. List of primers used in this study.

Names	Sequences (5’ to 3’)	Purposes
PtPYRL1-F	GGGCCCGGGATGACTGACCCAGCACAACAAGAAC	*PtPYRL1* Gene Cloning
PtPYRL1-R	GGGGTCGACTTATTTACCGTCACCGTCACGAGC	
PtPYRL5-F	GGGGGATCCATGCCTGCATCACTACAGCTCCAG	*PtPYRL5* Gene Cloning
PtPYRL5-R	GGGGTCGACTCATGATGATGTAGAAATCTGGGCAT	
PtPYRL1-RT-F	CCCACCACGTCACAATCCCACCTAG	*PtPYRL1* RT-PCR and qRT-PCR
PtPYRL1-RT-R	GCCACGCTGCAGCTCTTGATGAAG	
PtPYRL5-RT-F	GCCGCCATCCCTACCACAACAAC	*PtPYRL5* RT-PCR and qRT-PCR
PtPYRL5-RT-R	CGGTGGAGACTGGTGCATTGATGG	
EF1β-RT-F	GACAAGAAGGCAGCGGAGGAGAG	*EF1β* RT-PCR and qRT-PCR
EF1β-RT-R	CAATGAGGGAATCCACTGACACAAG	
ACTIN2-RT-F	ACTCTCCCGCTATGTATGTCGCC	*ACTIN2* RT-PCR and qRT-PCR
ACTIN2-RT-R	ATTTCCCGCTCTGCTGTTGTGGT	
PtPYRL1-F	GGGCCCGGGATGACTGACCCAGCACAACAAGAAC	PtPYRL1 GFP Construction
PtPYRL1-R2	GGGCTCGAGTTTACCGTCACCGTCACGAGC	
PtPYRL5-F	GGGGGATCCATGCCTGCATCACTACAGCTCCAG	PtPYRL5 GFP Construction
PtPYRL5-R2	GGGCTCGAGTGATGATGTAGAAATCTGGGCATTCC	
PtABI1B-F	GGGGAATTCATGGAGGAGATGTATCCGGCG	PtABI1B GFP Construction
PtABI1B-R	GGGGGATCCCGTGTTTTGGTTTTGAACTTCCTTT	
PtSnRK2.11-F	GGGGAATTCATGGATAGATCAGTGATGACAGTGGG	PtSnRK2.11 GFP Construction
PtSnRK2.11-R	GGGGGATCCCGCATTGCATATACTATCTCTCCACTG	
PtPYRL1-F3	GGGCCCGGGTATGACTGACCCAGCACAACAAG	PtPYRL1 Yeast two-hybrid
PtPYRL1-R3	GGGGAGCTCGTTATTTACCGTCACCGTCACGAG	
PtPYRL5-F3	GGAATTCATGCCTGCATCACTACAGCT	PtPYRL5 Yeast two-hybrid
PtPYRL5-R3	CGCGGATCCTCATGATGATGTAGAAATCTGGG	
PtABI1B-F	GGGGAATTCATGGAGGAGATGTATCCGGCG	PtABI1B Yeast two-hybrid
PtABI1B-R2	GGGGGATCCCTCATGTTTTGGTTTTGAACTTCCTTT	
PtSnRK2.11-F	GGGGAATTCATGGATAGATCAGTGATGACAGTGGG	PtSnRK2.11 Yeast two-hybrid
PtSnRK2.11-R2	GGGGGATCCCTCACATTGCATATACTATCTCTCCACTG	
P35S	TGACGCACAATCCCACTATC	General Use
